# Supplementary material for: Memorization bias impacts modeling of alternative conformational states of solute carrier membrane proteins with methods from deep learning
Source: PLoS Comput Biol. 2025 Oct 17;21(10):e1013590. doi: 10.1371/journal.pcbi.1013590 (PMC12551959; doi:10.1371/journal.pcbi.1013590)
Supplement: S1 Table — (DOCX) [file pcbi.1013590.s001.docx]

**S1 Table.** Assessment of modeling alternative states of SLC proteins with AF2.

**
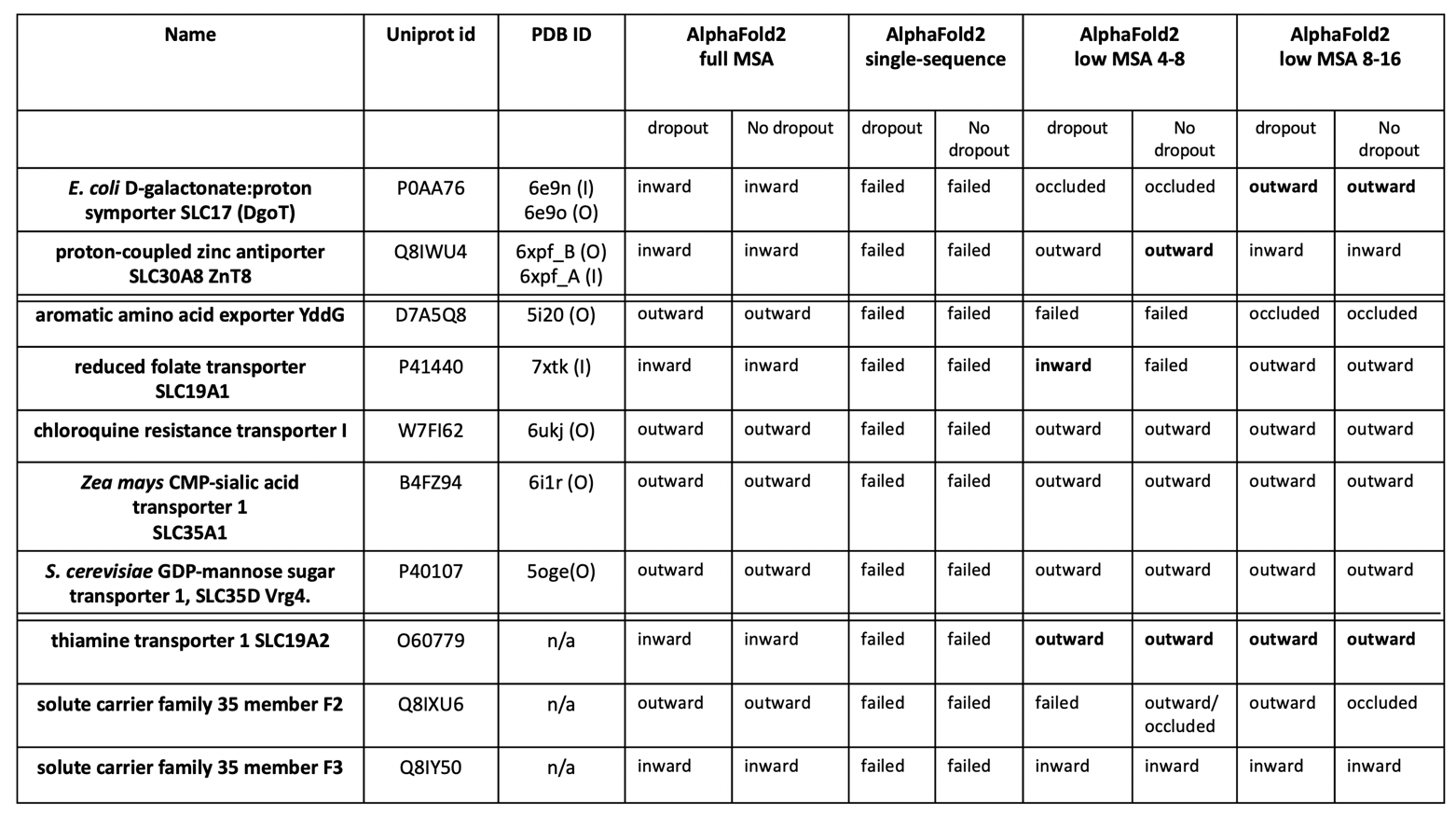
**

The outcomes “inward”, “outward”, and “occluded” indicate the conformational state observed, and the outcome “failed” indicates that a reasonable structure consistent with EC contact maps and/or experimental structures was not obtained. Results in which the alternative conformational state was generated are indicated in bold font.
