# Supplementary material for: Memorization bias impacts modeling of alternative conformational states of solute carrier membrane proteins with methods from deep learning
Source: PLoS Comput Biol. 2025 Oct 17;21(10):e1013590. doi: 10.1371/journal.pcbi.1013590 (PMC12551959; doi:10.1371/journal.pcbi.1013590)
Supplement: S2 Table — (DOCX) [file pcbi.1013590.s002.docx]

**S2 Table**. Assessment of successful generation of alternative states of SLC proteins from flipped sequence using *ESMfold* vs *AlphaFold2*.


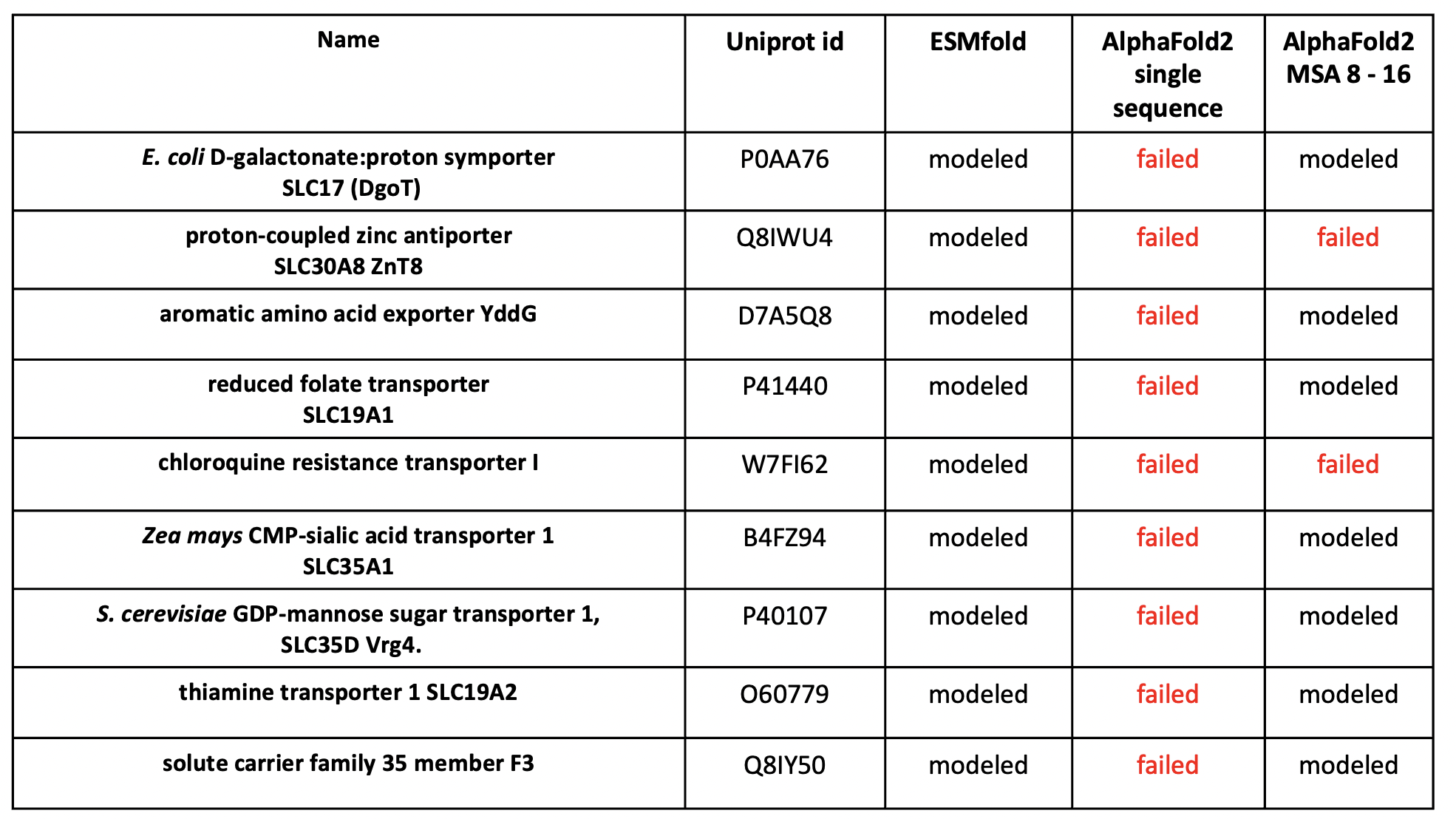


For each of these 9 SLC proteins, a “flipped sequence” was used to generate a template for modeling the alternative conformation state, as outlined in main text **Fig. 1**, using either *ESMfold* or *AF2*. The outcome “modeled” indicates that the result was a folded protein with a structure generally similar to the alternative inward- or outward-open conformational state; while “failed” (in red font) indicates that a reasonable structure was not obtained. Both methods were initially performed using a single-sequence as input; in this case the *ESMfold* models were alternative conformational states as expected, but the AF2 modeling based on the “flipped sequence” failed for all 9 proteins. The *AF2* modeling from the “flipped sequence” was then repeated with MSA max of 8 to 16, in which case 7 of the 9 systems provided reasonable structures for the alternative state.
