# Supplementary material for: Memorization bias impacts modeling of alternative conformational states of solute carrier membrane proteins with methods from deep learning
Source: PLoS Comput Biol. 2025 Oct 17;21(10):e1013590. doi: 10.1371/journal.pcbi.1013590 (PMC12551959; doi:10.1371/journal.pcbi.1013590)
Supplement: S3 Table — (DOCX) [file pcbi.1013590.s003.docx]

**S3 Table**. Modeling of both outward-open and inward-open states of pseudo-symmetric SLC proteins


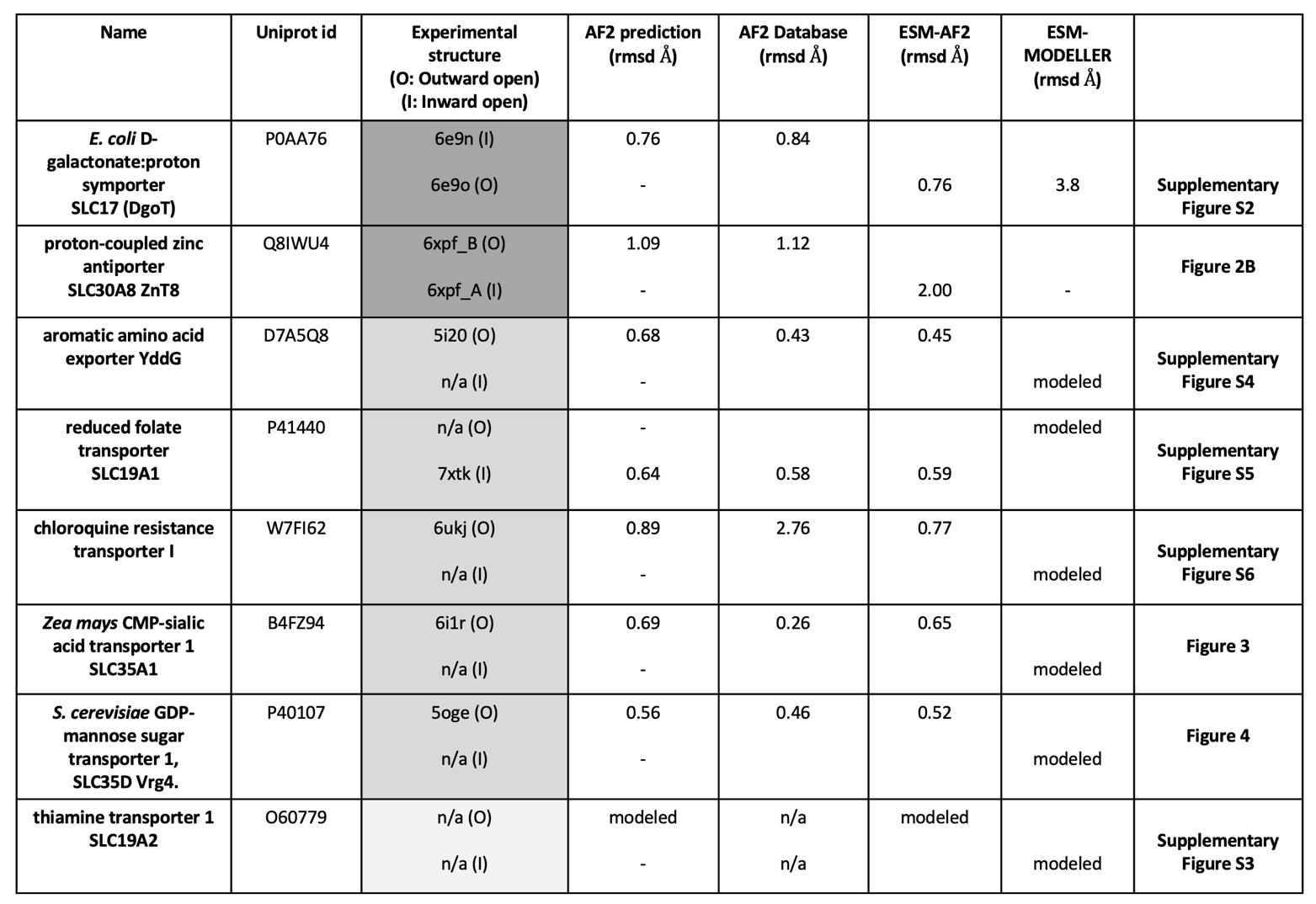


For each SLC protein, conventional AF2 modeling provided either an inward-open (I) or outward-open (O) state for which the backbone root-mean-squared deviation to an available X-ray crystal or cryoEM structure is listed. Shading designates where both states (dk grey), neither state (med grey), and one state (lt gray) were available in the PDB at time of AF2 training. The alternative outward-open or inward-open conformation was then generated with the ESM-AF2 or ESM-MODELLER protocol. For these models of alternative conformational states, backbone rmsd’s are reported where experimental structures are available. Models for each protein available in the AlphaFold2 data base are indicated along with backbone rmsd’s to the most similar experimental structure. The outcome “modeled” indicates that the result was a folded protein with a structure generally similar to the alternative inward- or outward-open conformational state, n/a – an experimental structure is not available.
