# Supplementary material for: Memorization bias impacts modeling of alternative conformational states of solute carrier membrane proteins with methods from deep learning
Source: PLoS Comput Biol. 2025 Oct 17;21(10):e1013590. doi: 10.1371/journal.pcbi.1013590 (PMC12551959; doi:10.1371/journal.pcbi.1013590)
Supplement: S4 Table — (DOCX) [file pcbi.1013590.s004.docx]

**S4 Table**. Comparing bias of ESF-AF2, ESM-AF3, and ESM-MODELLER protocols in flipping the conformational state of SLC proteins.

| **Name** | **ESM-modeled**  **flipped-sequence template** | **ESM-AF2** | **ESM-AF3** | **ESM-MODELLER** |
| --- | --- | --- | --- | --- |
| ***E. coli* D-galactonate:proton symporter**  **SLC17 (DgoT)** | outward-open | outward-open | outward-open | outward-open |
| **proton-coupled zinc antiporter**  **SLC30A8 ZnT8** | inward-open | inward-open | inward-open | inward-open |
| **aromatic amino acid exporter YddG** | inward-open | outward-open | outward open | inward-open |
| **reduced folate transporter**  **SLC19A1** | outward-open | inward-open | outward-open | outward-open |
| **chloroquine resistance transporter I** | inward-open | outward-open | outward-open | inward-open |
| ***Zea mays* CMP-sialic acid transporter 1**  **SLC35A1** | inward-open | outward-open | outward-open | inward-open |
| ***S. cerevisiae* GDP-mannose sugar transporter 1,**  **SLC35D Vrg4.** | inward-open | outward-open | outward-open | inward-open |
| **thiamine transporter 1 SLC19A2** | outward-open | inward-open | outward-open | outward-open |
| **solute carrier family member SLC35 F2** | inward-open | inward-open | outward-open | inward-open |

Results shown in red font are cases where the AI inference flips the state relative to the template's structural state.

**.**
