# Supplementary material for: Memorization bias impacts modeling of alternative conformational states of solute carrier membrane proteins with methods from deep learning
Source: PLoS Comput Biol. 2025 Oct 17;21(10):e1013590. doi: 10.1371/journal.pcbi.1013590 (PMC12551959; doi:10.1371/journal.pcbi.1013590)
Supplement: S1 Fig — (DOCX) [file pcbi.1013590.s005.docx]

**
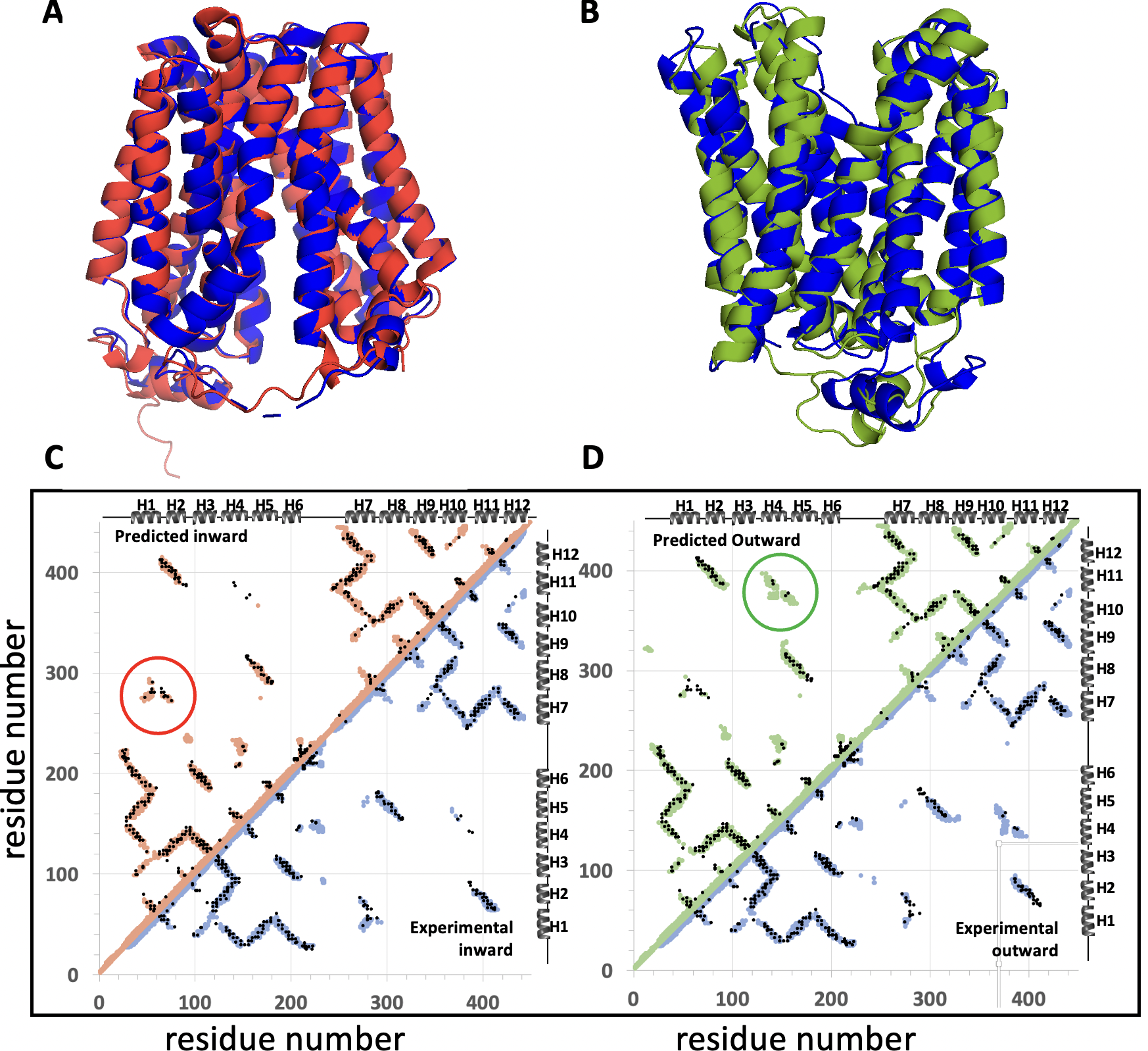
**

**S1 Fig. Validation of ESM-AF2 protocol using an SLC protein with both outward- and inward-open experimental structures.** (A) Superposition of the inward-open conformational state of *E. coli* D-galactonate:proton symporter (SLC17, DgoT) modeled using the ESM-AF2 protocol (red) with the experimentally-determined inward-open structure PDB id 6e9n (blue). (B) Superposition of the outward-open state modeled using the ESM-AF2 protocol (green) with the experimentally-determined inward-open structure PDB id 6e9o (blue). (C) Contact maps for the inward-open state of DgoT predicted by ESM-AF2 (red circles), observed in the cryoEM experimental structure (blue circles ), and predicted by EC analysis (black dots). (D) Contact maps for the outward-open state of DgoT predicted by ESM-AF2 (green circles), observed in the cryoEM experimental structure (blue circles ), and predicted by EC analysis (black dots). In this example, only a few EC-based contacts between helices H4 and H11 and helices H5 and H10 (circled in green in panel D) and between H1 and H7 (circled in red in panel C) distinguish the inward- from outward-open structures.
