# Supplementary material for: Memorization bias impacts modeling of alternative conformational states of solute carrier membrane proteins with methods from deep learning
Source: PLoS Comput Biol. 2025 Oct 17;21(10):e1013590. doi: 10.1371/journal.pcbi.1013590 (PMC12551959; doi:10.1371/journal.pcbi.1013590)
Supplement: S2 Fig — (DOCX) [file pcbi.1013590.s006.docx]

**
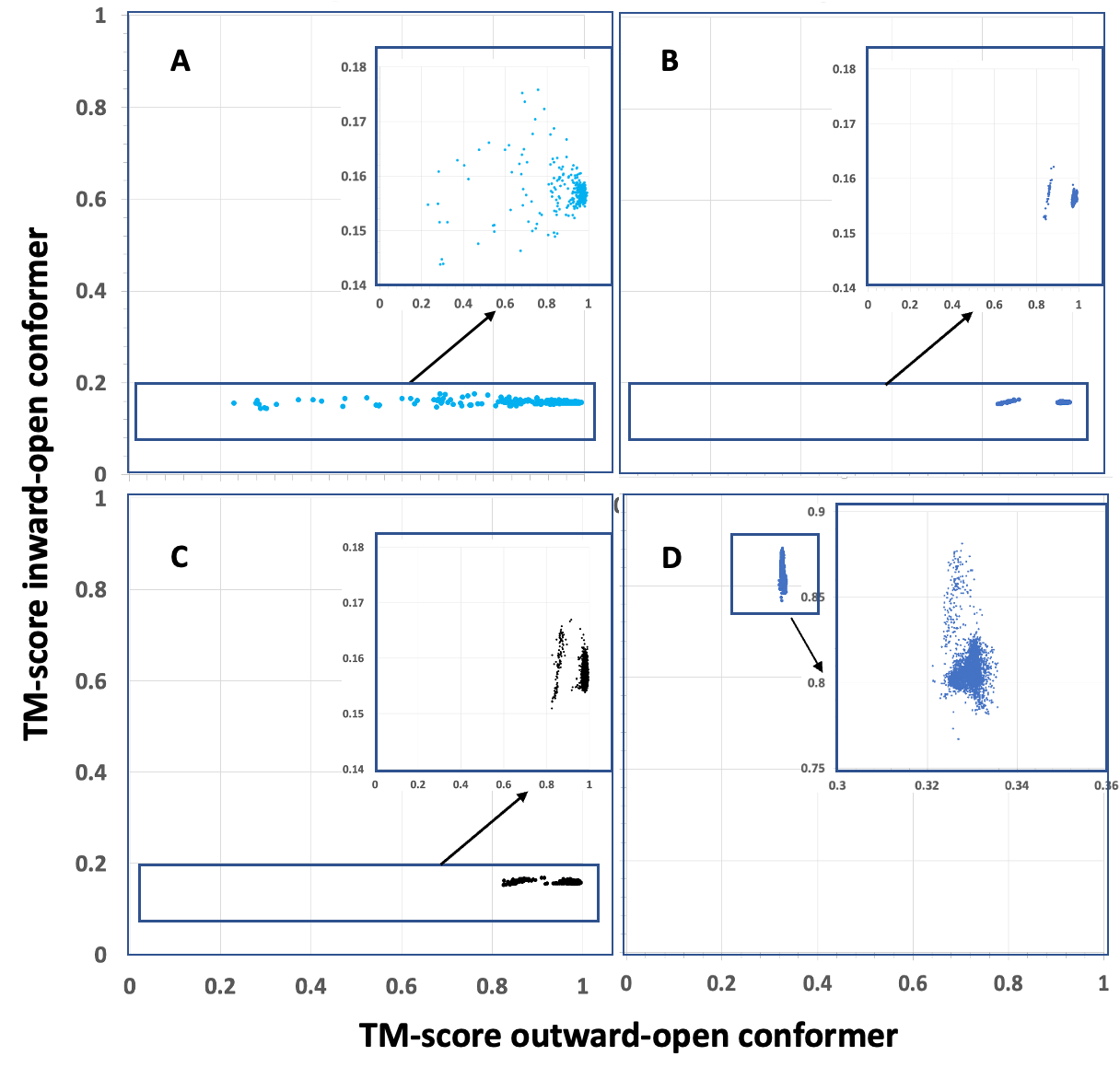
**

**S2 Fig. Conformational states of SLC35F2 modeled using various enhanced or massive sampling protocols.** (A) *AlphaFold-alt* (480 models), (B) *AlphaFold-sample* (3,000 models), and (C) *AlphaFold-sample2* (3,000 models); the resulting models were compared with the outward-open and inward-open models from the standard AF2 (for outward-open) and our ESM-AF2 protocol (for inward-open). None of these three methods modeled the alternative “inward-open” conformational state. (D) Massive sampling with *AF-sample2* (3,000 models) using a “flipped” protein sequence generated only inward-open conformations for SLC35F2 can provide an inward-open structure of SLC35F2. In each panel, the inset shows an expanded region of the plot. In all cases, no models were observed outside of the boxed regions. All models have <pLDDT > > 70.
