# Supplementary material for: Memorization bias impacts modeling of alternative conformational states of solute carrier membrane proteins with methods from deep learning
Source: PLoS Comput Biol. 2025 Oct 17;21(10):e1013590. doi: 10.1371/journal.pcbi.1013590 (PMC12551959; doi:10.1371/journal.pcbi.1013590)
Supplement: S5 Fig — (DOCX) [file pcbi.1013590.s009.docx]

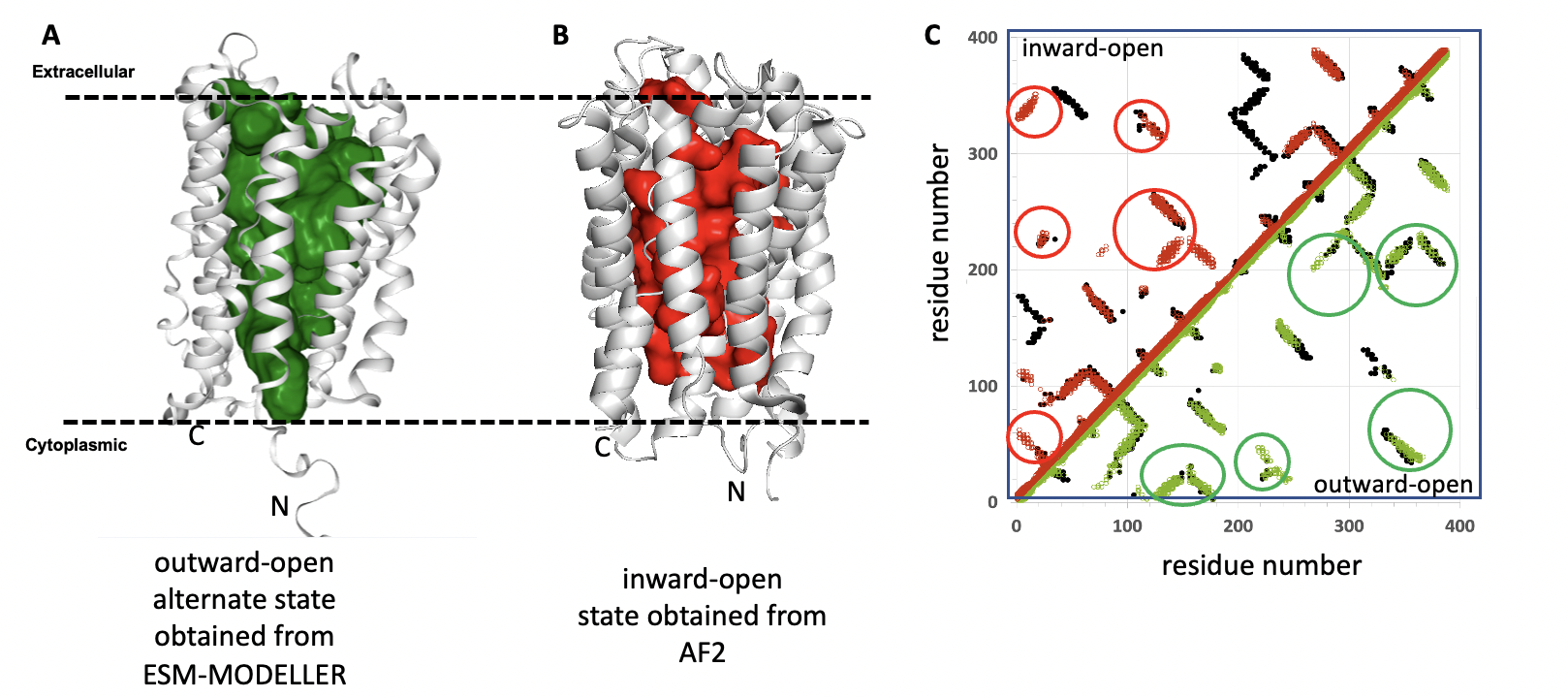


**S5 Fig. Reduced folate transporter (SLC19A1).** (A) The outward-open conformer was generated using the ESM-MODELLER method. (B) AF2 models the inward-open state. (C) The outward-open conformer contact map (green, lower diagonal), and inward-open conformer contact map (red, upper diagonal), generated using Cα-Cα cutoff of 10 $Å,$ are superposed on the symmetric EC-predicted contact map (black). ECs unique to each state are indicated with green and red circles, respectively.
