# Supplementary material for: Memorization bias impacts modeling of alternative conformational states of solute carrier membrane proteins with methods from deep learning
Source: PLoS Comput Biol. 2025 Oct 17;21(10):e1013590. doi: 10.1371/journal.pcbi.1013590 (PMC12551959; doi:10.1371/journal.pcbi.1013590)
Supplement: S7 Fig — (DOCX) [file pcbi.1013590.s011.docx]

We created an alternative set of relevant Main Text and Supplementary Figures by contrast adjustment, and reviewed these alternative figures with an Online Color Blindness Simulator (DaltonLens https://daltonlens.org/colorblindness-simulator) using the method of Brettel, Viénot & Mollon (1997).  These adjusted images are provided here as **Supplementary Color Adjusted Figures.**

Brettel H, Viénot F, Mollon JD. Computerized simulation of color appearance for dichromats. J Opt Soc Am A Opt Image Sci Vis. 1997 Oct;14(10):2647-55. doi: 10.1364/josaa.14.002647. PMID: 9316278.

**
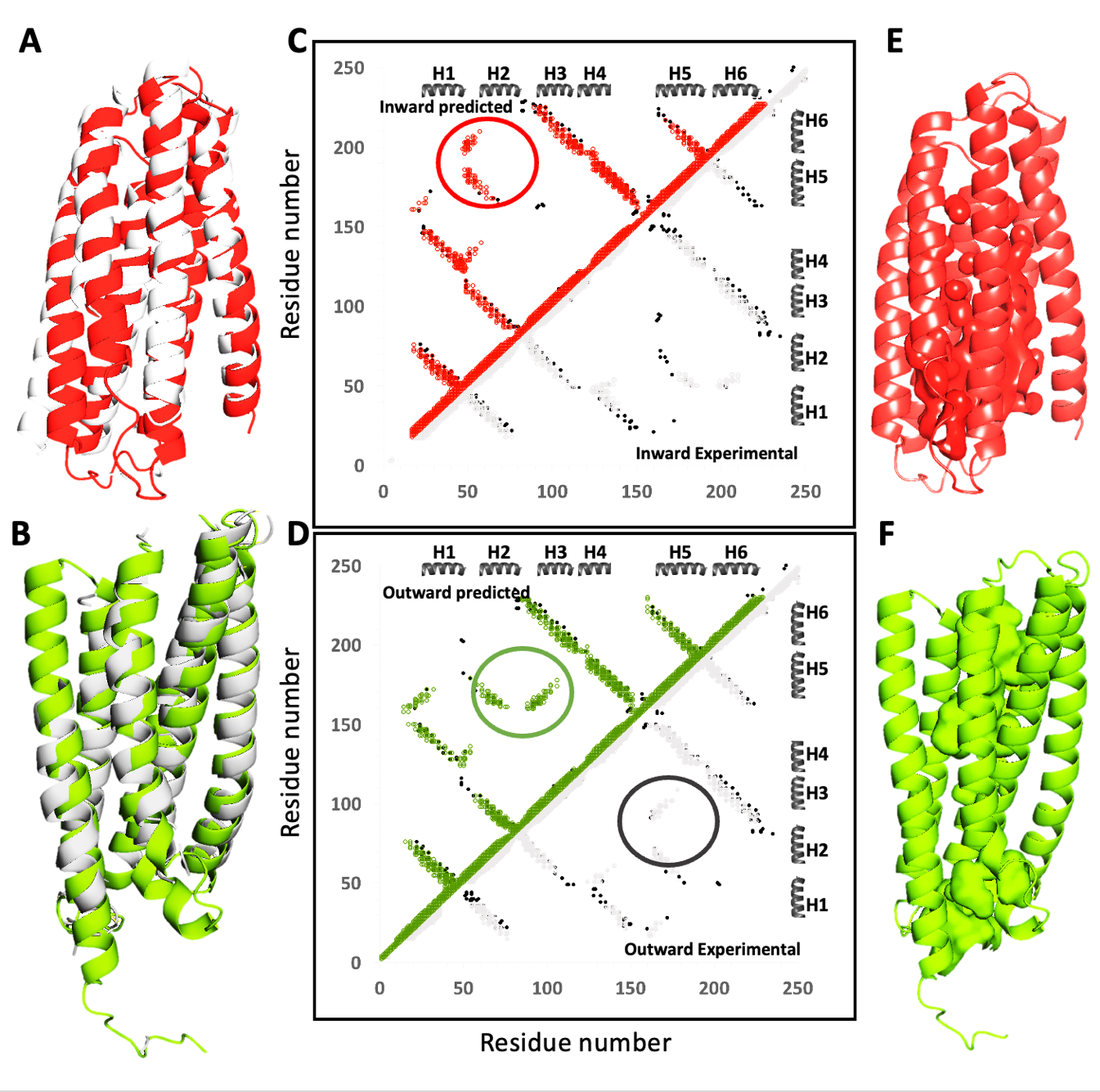
**

**S7 Fig. Color-bind adjusted version of Fig. 2.**
