# Supplementary figures and images for: Memorization bias impacts modeling of alternative conformational states of solute carrier membrane proteins with methods from deep learning
Source: PLoS Comput Biol. 2025 Oct 17;21(10):e1013590. doi: 10.1371/journal.pcbi.1013590 (PMC12551959; doi:10.1371/journal.pcbi.1013590)

**
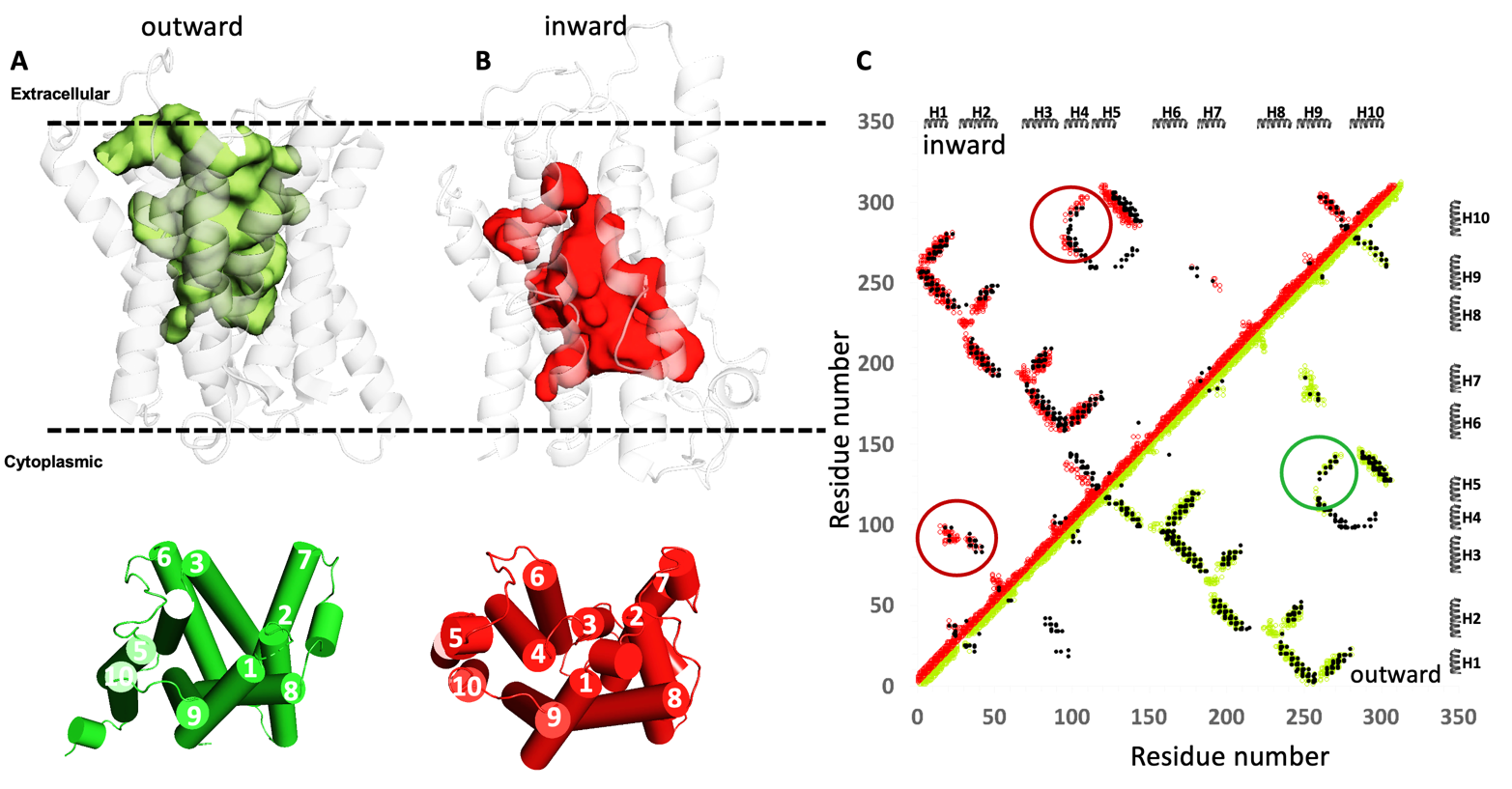
**

**Fig S8. Color-bind adjusted version of Fig. 3.**

Supplement: S8 Fig — (DOCX) [file pcbi.1013590.s012.docx]

**
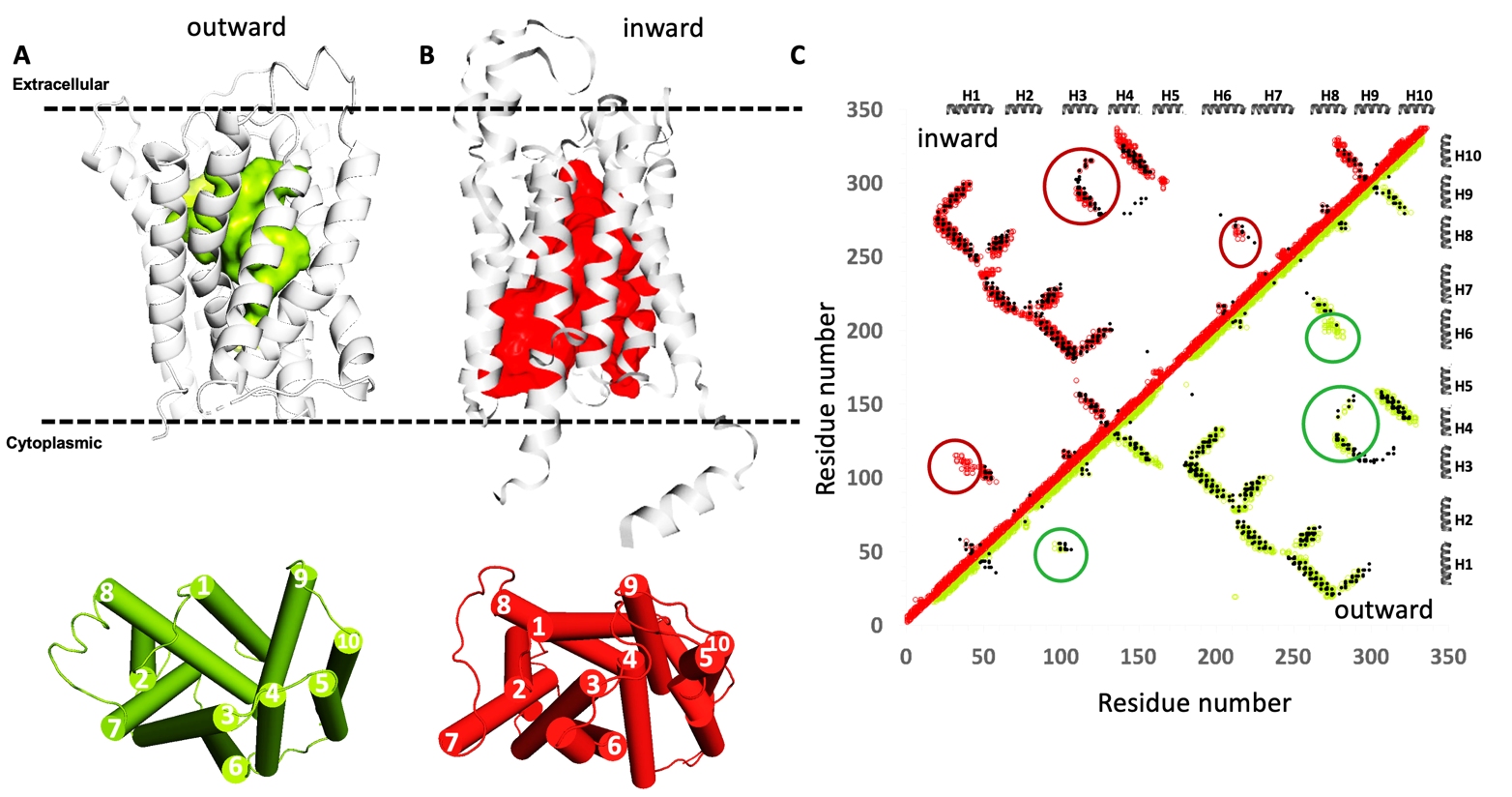
**

**S9 Fig. Color-bind adjusted version of Fig. 4.**

Supplement: S9 Fig — (DOCX) [file pcbi.1013590.s013.docx]

**
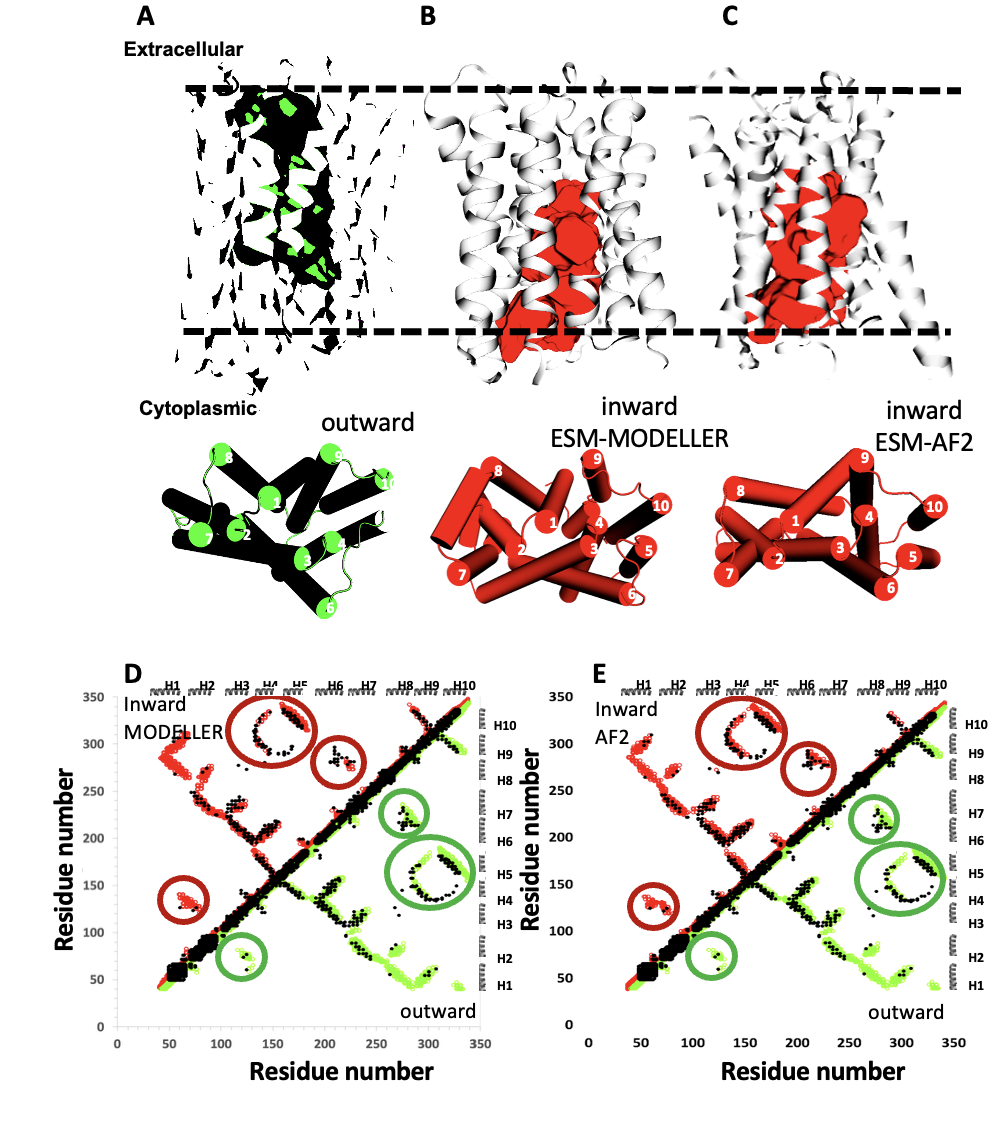
**

**S10 Fig. Color-bind adjusted version of Fig. 5.**

Supplement: S10 Fig — (DOCX) [file pcbi.1013590.s014.docx]

**
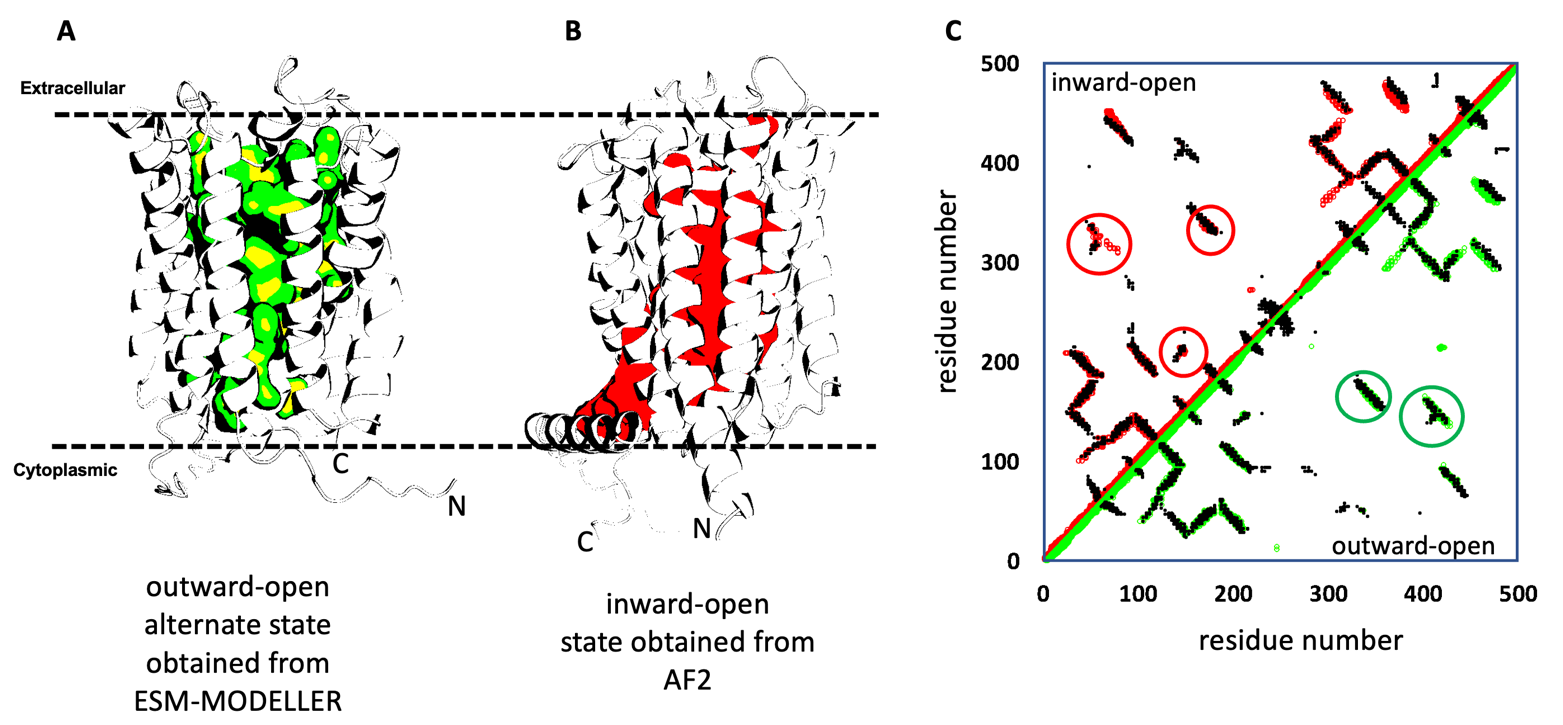
**

**S11 Fig. Color-bind adjusted version of S3 Fig.**

Supplement: S11 Fig — Color-bind adjusted version of S3 Fig. (DOCX) [file pcbi.1013590.s015.docx]

**
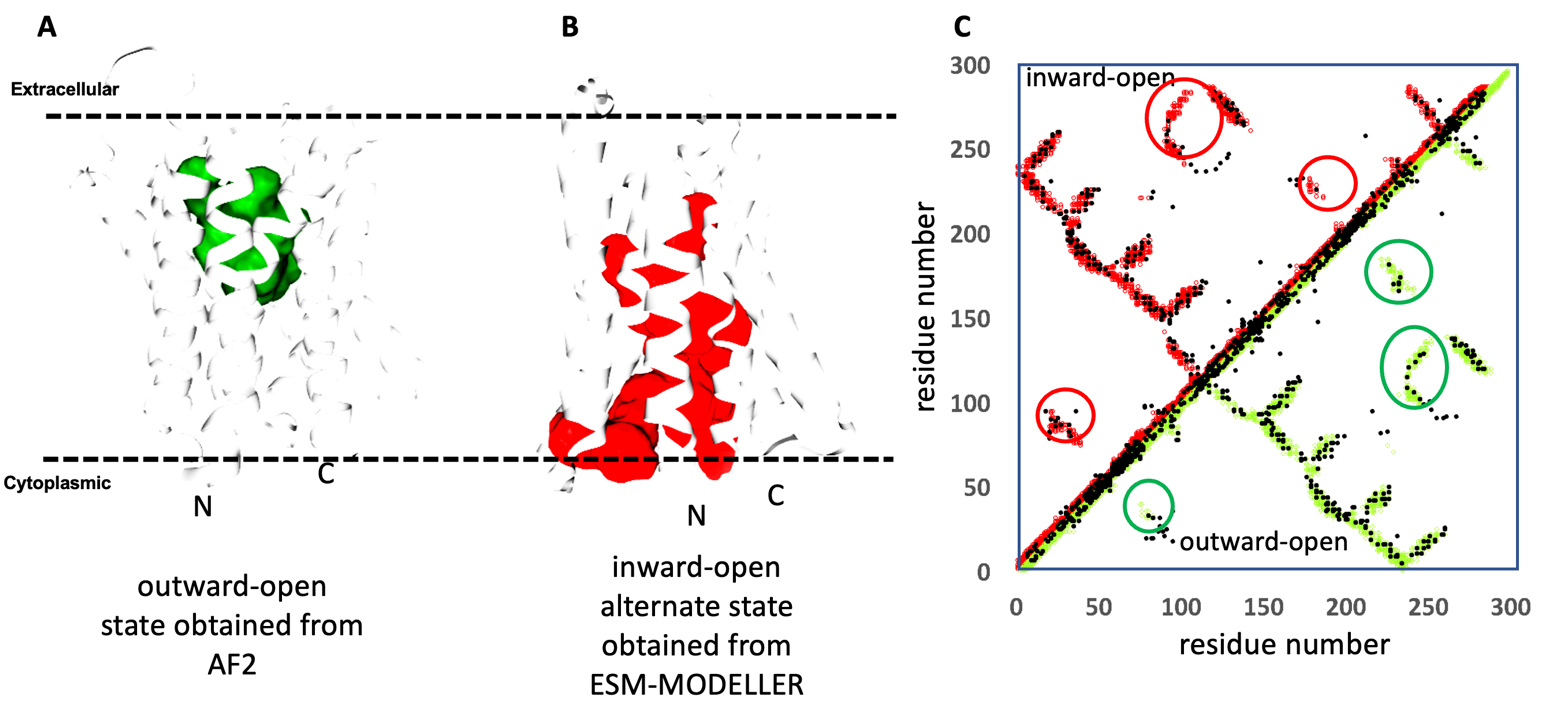
**

**S12 Fig. Color-bind adjusted version of S4 Fig.**

Supplement: S12 Fig — Color-bind adjusted version of S4 Fig. (DOCX) [file pcbi.1013590.s016.docx]

**
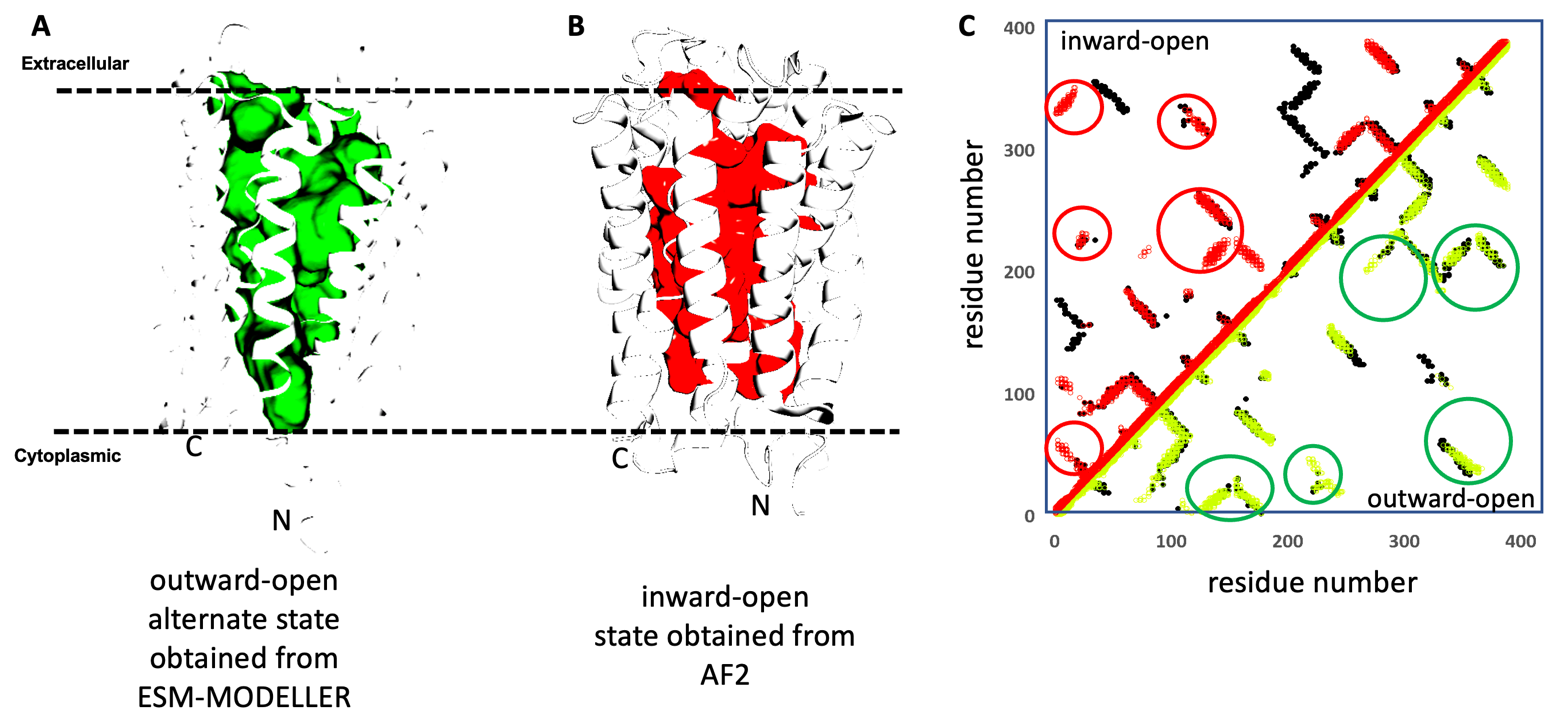
**

**S13 Fig. Color-bind adjusted version of S5 Fig.**

Supplement: S13 Fig — Color-bind adjusted version of S5 Fig. (DOCX) [file pcbi.1013590.s017.docx]

**
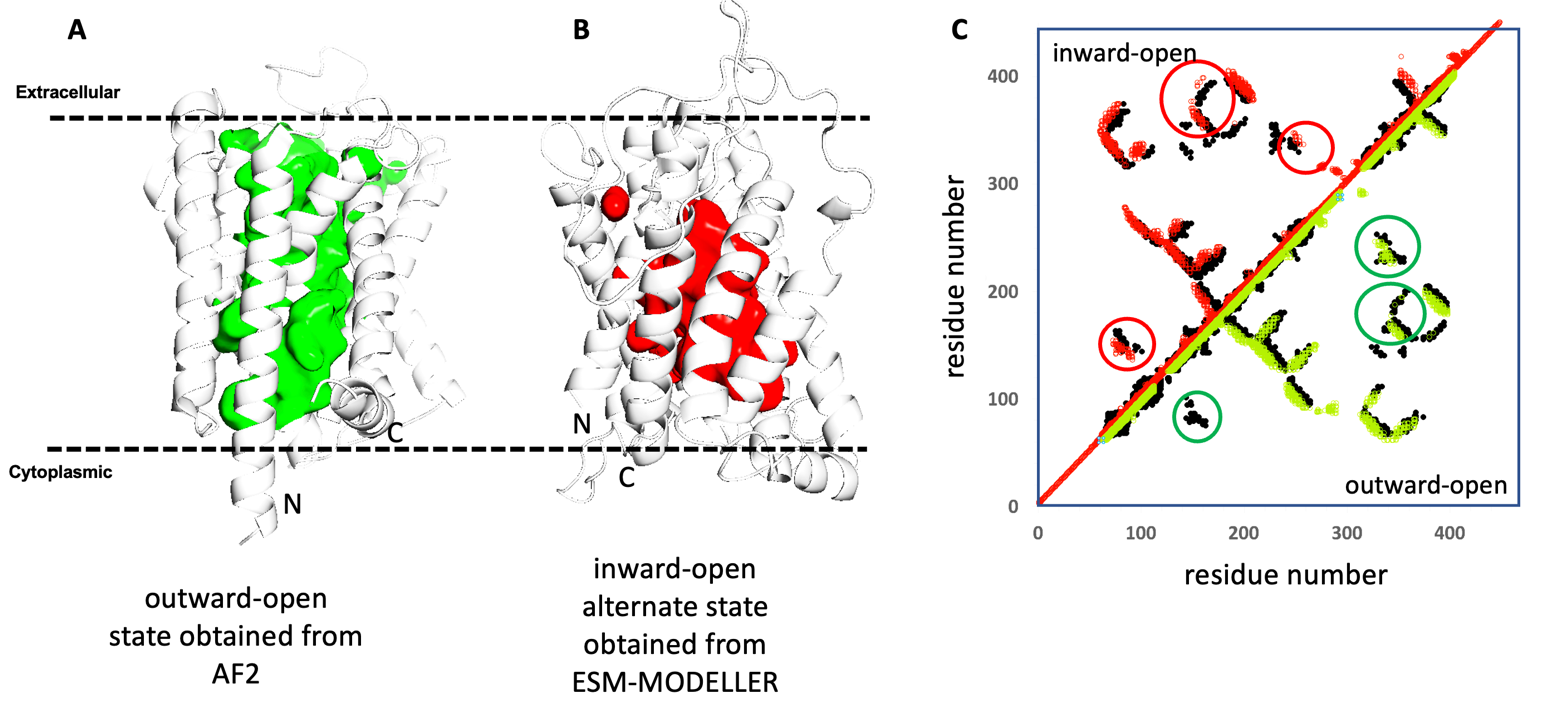
**

**S14 Fig. Color-bind adjusted version of S6 Fig.**

Supplement: S14 Fig — Color-bind adjusted version of S6 Fig. (DOCX) [file pcbi.1013590.s018.docx]
