# Supplementary material for: Memorization bias impacts modeling of alternative conformational states of solute carrier membrane proteins with methods from deep learning
Source: PLoS Comput Biol. 2025 Oct 17;21(10):e1013590. doi: 10.1371/journal.pcbi.1013590 (PMC12551959; doi:10.1371/journal.pcbi.1013590)
Supplement: Attachment — Submitted filename: Reviewers Comments.Responses.vF.pdf [file pcbi.1013590.s020.pdf]

RESPONSES TO REVIEWER COMMENTS

We have addressed all of the issues raised by each of three reviewers. We provide for each **Reviewer Comment** a **Response** and summary of how the text has been revised to address the comment. The most challenging issue, which was the reason for our long delay in resubmission, was the concern raised about red/green color blindness (**Reviewer 3, Comment 3.5**), which is now fully addressed by providing alternative images readable by all three forms of severe color blindness as **Supplementary Color-blind Adjusted Figures.**

Reviewers' comments:

Reviewer #1: Swapna et al. used a template-based ESM modelling approach to obtain the alternate conformational states of SLC proteins. The authors also compared several ML based approaches such as AF2, AF3, ESM; highlighting the current limitations of these ML methods in modeling multiple conformational states of SLC proteins. The obtained structures were validated by using the EC data.

Comment 1.1. The authors claimed to validate these structures using EC data as “experimental validation”, which I find surprising. Perhaps “validation” is more appropriate than “experimental validation”.

Response 1.1. In response to this comment, we have replaced "experimental validation" with "validation" throughout the text. However, we have also added this point on page 13

> In this study, alternative conformational states of SLC proteins were validated against contact predictions based on evolutionary co-variance (ECs). ECs are an interpretation of experimental protein sequence data, and hence this validation is effectively against experimental data. However, this validation is not as robust as validation against protein-specific experimental data such as X-ray crystallography, NMR, or cryoEM data.

The authors also highlighted the limitation of their high performing ESM-AF2 approach in modelling SLC proteins alternate states in several scenarios. The manuscript is well written and will be useful for researchers working on transmembrane proteins with multiple conformational states.

Minor points:

Page 13, line 3: “is it” should be “it is”

This has been corrected.

Page 13, 3rd paragraph: “Lazou al” should be “Lazou et al.”

This has been corrected.

Page 25, Fig. S3: The green and red circles seem to be flipped.

This has been corrected in **Supplementary Figure S3**.

Reviewer #2: Though AlphaFold has revolutionized protein structure prediction, it has limitations. Here, Swapna and colleagues show one of its limitations–and some workarounds–when modeling alternative conformations of SLC Membrane proteins. The observations and approaches presented are useful, and I applaud the authors’ use of ECs to check the plausibility of the alternative conformations they generate. This work is solid and suitable for publication after a few minor concerns are addressed:

Comment 2.1. On Page 10, it is claimed that AF3 does not support template-directed modeling. What does this mean? AF3 uses templates in default mode, and it seems the input files could be modified to include templates of interest.

Response 2.1. Although the AlphaFold3 server - which is broadly available to the scientific community - does not support template-based inference, Reviewer 2 is correct to point out that the underlying AI does support templates as input, and this template-based inference can be done with a locally-installed instance of AlphaFold3. In order to fully address this concern of Reviewer 2, we have used a locally-installed instance of AF3 which allows template-directed modeling to address this point. The following text has been revised on page 10,

> Modeling was also attempted using AF3 [1] without templates. Only the outward-open conformational state of SLC35 was returned by AF3. Hence, even without a state-specific structure in their training sets, AF2 (and AF3) are biased towards the outward-open state of SLC35F2.

And to describe the templated-based modeling results with AF3, we added the following results on page 13, along with **Supplementary Table S4**.

> We also tested if the bias of AF2 to flip the conformational state of the ESM-generated template structure is also observed for AF3. This study is summarized in **Supplementary Table S4**. While the server version of AF3 does not support user-provided templates, the study could be done using a locally-installed version of AF3 with full MSAs generated with jackHMMR [2]. The same results were obtained using MSAs generated by MMseqs [3]. Considering the 6 proteins for which the ESM-AF2 protocol flipped the states of the input template, for two of these, reduced folate transporter SLC19A1 and thiamine transporter 1 SLC19A2,

the AF3 protocol did not flip the state from the template; the overall ESM-AF3 protocol thus provided both states. However, for four others, aromatic amino acid exporter YddG, chloroquine resistance transporter I, *Zea mays* CMP-sialic acid transporter 1 SLC35A1, and *S. cerevisiae* GDP-mannose sugar transporter 1 SLC35D Vrg4, the AF3 protocol, like the AF2 protocol, did flip the state from the template; and the overall ESM-AF3 protocol did not provide the alternative state. For the remaining two systems tested, both the ESM-AF2 and ASM-AF3 protocols were successfully guided by the ESM-generated template to successfully model the alterative state. Similar results were obtained using shallow MSAs. On the other hand, for SLC35F2 using AF3 [1] with an inward-facing template generated by *ESMfold* returned only outward-facing models. Hence, the impact of training memorization on both the ESM-AF2 and ESM-AF3 protocols is highly variable and depends on multiple factors including the inference engine used and the nature and depth of the input MSA. These results demonstrate the importance of cross-validating models against EC or (if available) sample-specific experimental data.

Comment 2.2. On Page 5, it says that AlphaFold-alt was run generating 30 conformations for sequence depths ranging from 16-32. Was it also run to generate MSAs with 4-8 sequences specified in Table S1? The Methods could be more clear on this point.

Response 2.2. Thank you for noting this detail. In the results summarized in **Suppl Table S1** we also used 4-8, or even with a single sequence. This is now clarified on page 5.

Comment 2.3. Faezov and Dunbrack also used templates to bias AlphaFold2 to generate models of alternative conformations of protein kinases (https://www.biorxiv.org/content/10.1101/2023.07.21.550125v2). Although they used a somewhat different approach than presented here, their work should be cited.

Response 2.3. Thank you for this citation. We were aware of this work but did not cite it as it is Preprint. In any case, we have now added this citation it to the Introduction.

Comment 2.4. The manuscript seems to suggest that memorization occurs only when structures with the exact sequence being tested were in the database prior to training. This may be too narrow of a definition. AlphaFold can associate memorized structures with homologous sequences without evolutionary couplings (Chakravarty, et al. 2024). This is likely why, when CFold was trained (Bryant and Noé, 2024), all homologous alternative conformations were excluded from the training data. If one alternative structure is present in the training data, AF may associate its memorized structure with a homologous sequence without coevolutionary inference.

Response 2.4. Yes - we agree. Memorization can also result from homologous protein structures connected with the target protein through the MSA. We have clarified this in the text on page 14

> Memorization can also result from homologous protein structures connected with the target protein through the MSA [4].

Reviewer #3: This is a very interesting article providing not only quantitative insights into the limitations of machine-learning methods in structure prediction of dynamic proteins, but also providing a robust, consistent strategy to predict alternate states for membrane proteins with inverted topologies. These proteins have repeats within their sequences, that the authors reverse and feed into ESMfold to create a template with alternate conformation that can then be used for template-based modeling of the alternate state. This is a creative extension of the method that I and others have developed in the past, while avoiding the pitfalls of repeat-based sequence alignments and also leveraging the high-quality models that are created by machine learning methods. The paper is generally clearly written and should be of value for the structure prediction community, especially since the scripts are publicly available.

I only have a few comments for the author's consideration:

Comment 3.1. I wonder if "virtual flipped sequence" could/should be rephrased for clarity, perhaps "artificial" or "constructed" or "reordered" would be better terms. Essentially the repeated elements are swapped to create the new sequence.

Response 3.1. We like the term "flipped virtual sequence" (though it was the reviewer and her colleagues, not us, who first described this approach). However, when the term is first used on page 4 we now say

> ... reordered or " flipped virtual sequence".

Comment 3.2. I could not find mention of how the sequence repeats are identified so that they can be swapped: Where one structure is known, the repeat definitions identified by symmetry analysis could be used, e.g. from the Encompass database.

Response 3.2. We decided not to use the approach suggested, using the Encompass database, but we appreciate the reviewer's suggestion. The following text has been added in the Discussion section on page 14 to describe our experience.

> Symmetry in some integral membrane proteins such as SLC35F2 is easy to identify. Protein secondary structure and AF2 predictions indicate 10 helices connected by short loops. Chopping the sequence at the exact center of the loop

between symmetric halves and swapping the coordinates of the two halves orients the helices with inverted conformation that overlays with the EC contact maps. The flipped sequence when used as input into *ESMfold* provides a flipped conformer which can be used as a template for further predictions as described in the Methods section. For SLC proteins that have a long loop between their symmetric halves, chopping the sequence in the exact center fails to provide a flipped sequence that can be used to successfully generate a proper flipped conformer. In these cases, the long loop was replaced by an 8 - 12 residue polyglycine linker, and the split was made at the center of this linker. The resulting flipped sequence was sufficient for *ESMfold* to predict the flipped conformer. The contact maps generated for the flipped conformers are always compared with the EC-contact map. In cases where the match is not reflecting the contacts of the flipped state accurately, a few trials were made in the elimination of residues of the loop regions. It may also be possible to identify correct symmetry using repeat definitions identified by symmetry analyses available in the Encompass database [5].

Comment 3.3. An desirable extension of the method would (eventually) be to create complete conformational landscapes beyond the three major "inward-open", "occluded", "outward-open" states. This should be mentioned in the discussion.

Response 3.3. We have added the following text on page 14.

> Ultimately, it would be desirable to develop methods which can generate complete conformational landscapes beyond the three major "inward-open", "occluded", "outward-open" states [6, 7].

Comment 3.4. Based on the ideas of del Alamo et al, we have recently been successful in applying AF2.3 with sequence-downsampling to create an extensive ensemble for a heterodimeric mitochondrial pyruvate carrier, as an example (PMID: 40249800). Could perhaps providing two templates as simultaneous inputs allow ESMfold to explore all those states?

Response 3.4. This is a very interesting idea. The cited paper is very useful in this regard, and It is something we can consider in our future work.

Comment 3.5. Several figures rely on red and green for comparisons; colorblind-friendly alternatives should be created

Response 3.5. We have reviewed each figure in the Main Text and Supplementary Information to ensure that comparisons required to understand our study do not rely on red/green colors for comparisons. Although information for "inward-open" states are consistently colored red and information for "outward-open" states are consistently colored green, this information is also labeled in each subpanel with "inward-open" or "outward-open".

Here, for example, is **Suppl Figure S3**:

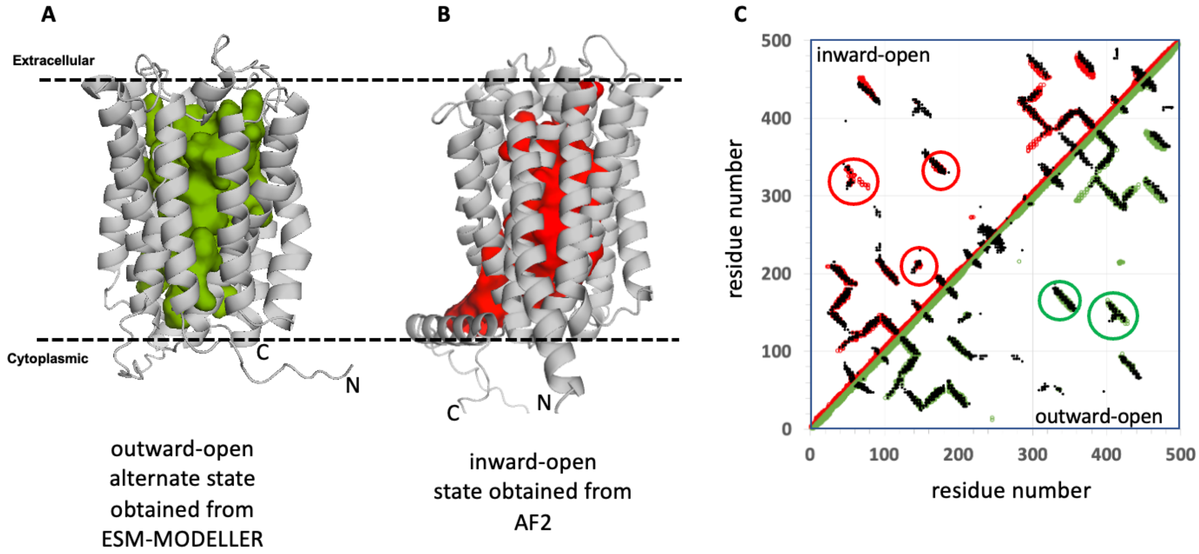

The information for the "outward-open" state is colored green in panel A, and in the lower diagonal of panel C. However, each of these panels is also labeled "outward-open". The information for the "inward-open" state is colored red in panel B, and in the upper diagonal of panel C. However, each of these panels is also labeled "inward-open". Hence, the reader does not need to distinguish green from red colors in order to fully understand the figure. The colored information is redundant with the labeled information. The same is true of all the other figures in the Main Text and Supplementary Material.

Accordingly, alternatives figures are not required.

However, in deference to Reviewer 3's concerns, we have created an alternative set of relevant Main Text and Supplementary Figures by contrast adjustment, and reviewed these alternative figures with an Online Color Blindness Simulator (DaltonLens https://daltonlens.org/colorblindness-simulator) using the method of Brettel, Viénot & Mollon [8]. These adjusted images are provided as **Supplementary Color Adjusted Figures.**

The method of Brettel, Viénot & Mollon [8] is reliable for the three common kinds of colorblindness; protanopia, deuteranopia, and tritanopia. Protanopia (severe red color blindness) results from deficient L-cones, leading to red-green color blindness in ~2% of males and ~0.04% of females. Deuteranopia (severe green color blindness) results from deficient M-cones, also leading to red-green color blindness in ~6% of males and ~0.4% of females. Tritanopia (severe blue color blindness) results from deficient S-cones, leading to blue-yellow color blindness in ~0.002% of males and ~0.001% of females. We also checked these results with other color blind simulators available on the DaltonLens web site.

As a representative example, here is the alternate version of **Suppl Fig. S3**, provided in **Supplementary Color Adjusted Figures** generated by adjusting the image contrast in PowerPoint (compare with figure above).

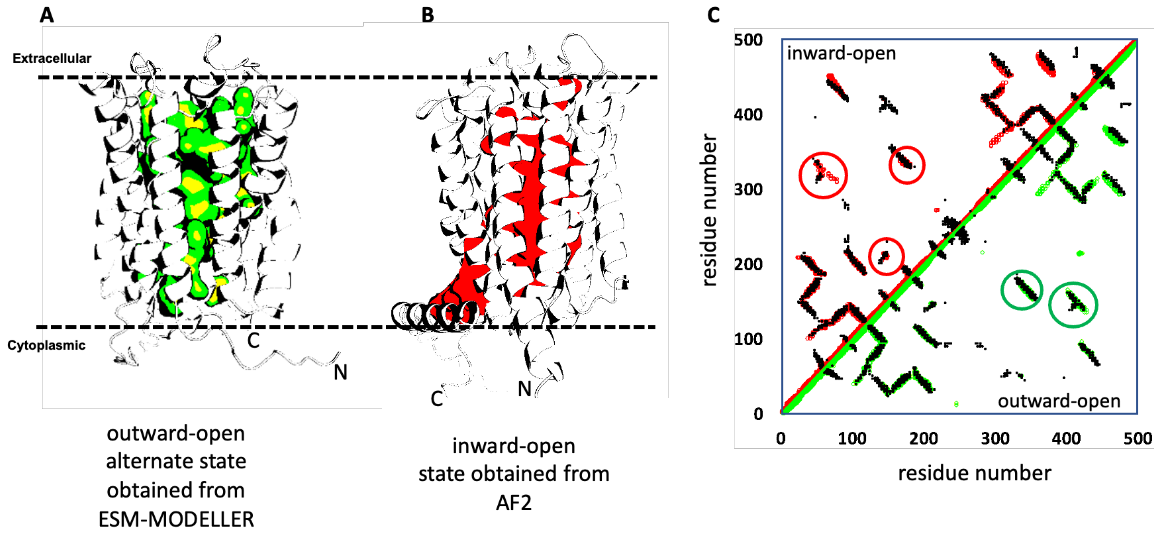

Note that the quality of this contrast-enhanced image is not as good at the images used in the Main Text and Supplementary Figures, and for this reason we did not replace the images in the paper with these images. Instead, we provide these contrast-enhanced images as **Supplementary Color-blind Adjusted Figures.**

The appearances of this image for individuals with 100% protanopia, deuteranopia, and tritanopia color blindness, respectively, simulated by the method of Brettel, Viénot & Mollon [8] are shown in the three panels below. Note that for individuals with less severe forms of color blindness, the discrimination between green and red information is even more distinct. Accordingly, individuals with even severe protanopia, deuteranopia, and tritanopia color blindness have full access to the information presented in our paper.

protanopia
(red blind)

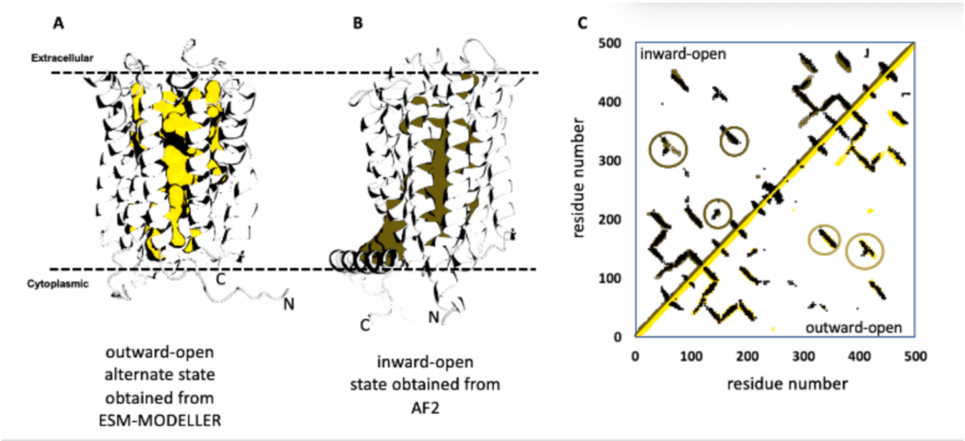

deutanopia
(green blind)

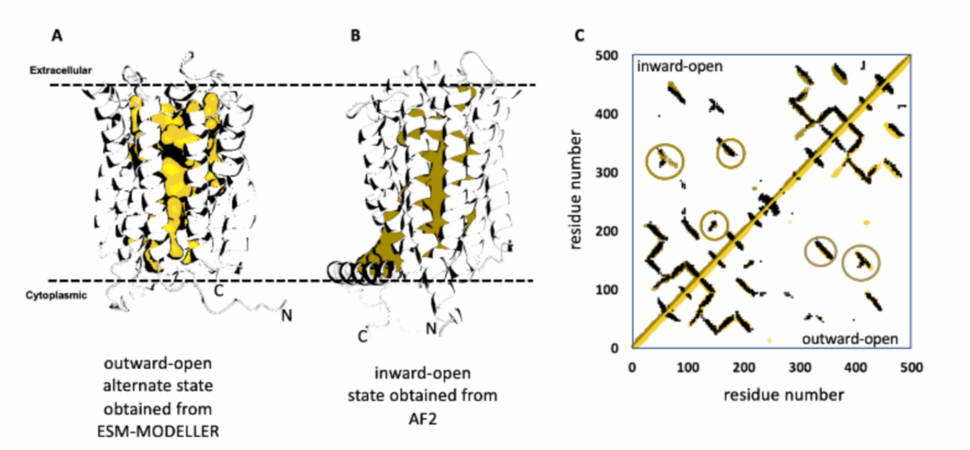

tritanopia
(blue blind)

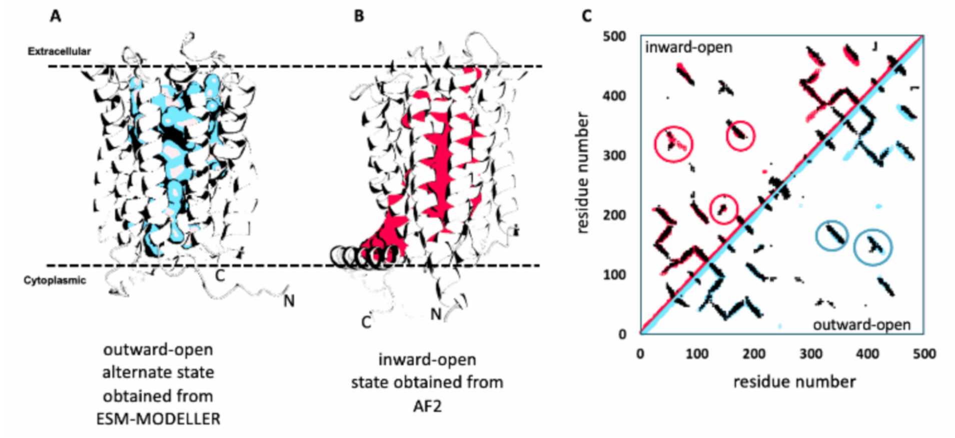

References for Response

1. Abramson J, Adler J, Dunger J, Evans R, Green T, Pritzel A, et al. Accurate structure prediction of biomolecular interactions with AlphaFold 3. Nature. 2024;630(8016):493-500. doi: 10.1038/s41586-024-07487-w.

2. Johnson LS, Eddy SR, Portugaly E. Hidden Markov model speed heuristic and iterative HMM search procedure. BMC Bioinformatics. 2010;11:431. Epub 20100818.

doi: 10.1186/1471-2105-11-431. PubMed PMID: 20718988; PubMed Central PMCID: PMCPMC2931519.
3. Hauser M, Steinegger M, Söding J. MMseqs software suite for fast and deep clustering and searching of large protein sequence sets. Bioinformatics. 2016;32(9):1323-30. doi: 10.1093/bioinformatics/btw006.
4. Chakravarty D, Schafer JW, Chen EA, Thole JF, Ronish LA, Lee M, et al. AlphaFold predictions of fold-switched conformations are driven by structure memorization. Nat Commun. 2024;15(1):7296. Epub 20240824. doi: 10.1038/s41467-024-51801-z. PubMed PMID: 39181864; PubMed Central PMCID: PMCPMC11344769.
5. Aleksandrova AA, Sarti E, Forrest LR. EncoMPASS: An encyclopedia of membrane proteins analyzed by structure and symmetry. Structure. 2024;32(4):492-504 e4. Epub 20240216. doi: 10.1016/j.str.2024.01.011. PubMed PMID: 38367624; PubMed Central PMCID: PMCPMC11251422.
6. Kalakoti Y, Wallner B. AFsample2: Predicting multiple conformations and ensembles with AlphaFold2. bioRxiv. 2024:2024.05.28.596195. doi: 10.1101/2024.05.28.596195.
7. Lewis S, Hempel T, Jimenez-Luna J, Gastegger M, Xie Y, Foong AYK, et al. Scalable emulation of protein equilibrium ensembles with generative deep learning. Science. 2025;389(6761):eadv9817. Epub 20250814. doi: 10.1126/science.adv9817. PubMed PMID: 40638710.
8. Brettel H, Viénot F, Mollon JD. Computerized simulation of color appearance for dichromats. J Opt Soc Am A Opt Image Sci Vis. 1997 Oct;14(10):2647-55. doi: 10.1364/josaa.14.002647. PMID: 9316278.
